# Kennedy Disease in Latin America: Two New Mexican Cases and a Systematic Review of Regional Reports

**DOI:** 10.7759/cureus.82861

**Published:** 2025-04-23

**Authors:** Carlos Alejandro Martínez-Zamora, Sergio Uriel Vidal-Michaca, Carmen Rubio, Miguel Ángel Ramírez-García

**Affiliations:** 1 Neurophysiology, National Institute of Neurology and Neurosurgery Manuel Velasco Suárez, Mexico City, MEX; 2 Social Service, Saint Luke School of Medicine, Mexico City, MEX; 3 Genetics, National Institute of Neurology and Neurosurgery Manuel Velasco Suárez, Mexico City, MEX

**Keywords:** kennedy's disease, latin america, spinal and bulbar muscular atrophy, systematic review, x-linked disorder

## Abstract

Spinal and bulbar muscular atrophy (SBMA), or Kennedy disease (KD), is a rare X-linked trinucleotide repeat disorder caused by cytosine, adenine, and guanine (CAG) expansions in the androgen receptor (AR) gene. KD affects lower motor neurons, leading to progressive muscle weakness, bulbar dysfunction, and endocrine symptoms such as gynecomastia. Diagnosis is challenging due to phenotypic overlap with other neurodegenerative diseases. This report aimed to describe two new Mexican cases of KD and to summarize all published cases of KD in Latin America through a systematic review (SR).

The SR search was performed using the Preferred Reporting Items for Systematic Reviews and Meta-Analyses (PRISMA) method in the Scielo, Lilacs, and Scopus databases, including cohort studies, case series, and case reports with molecular confirmation of SBMA. Clinical and molecular data were analyzed.

The review identified 23 Latin American SBMA cases. The median age of onset was 43 years (range: 21-66). The most common manifestations were weakness (95.7%; n = 22), tremor (65.2%; n = 15), and bulbar symptoms such as dysarthria (95.6%; n = 22) and dysphagia (91.3%; n = 21). Endocrine manifestations such as gynecomastia (73.9%; n = 17) and sexual dysfunction (56.5%; n = 13) were common. Electrophysiological findings confirmed lower motor neuron involvement, and molecular analysis revealed a median of 46.5 CAG repeats.

Despite its low frequency, SBMA remains underdiagnosed in Latin America, which may be due to limited awareness and clinical overlap with other pathologies. Molecular testing is crucial for diagnostic certainty. The review also highlights the need to evaluate multisystem involvement. Improved diagnostic protocols, genetic counseling, and increased awareness are needed for the timely detection and management of SBMA in Latin America.

## Introduction

Spinal and bulbar muscular atrophy (SBMA), the correct terminology for this disease, also known as Kennedy disease (KD), is a neurodegenerative genetic disorder that develops later in middle age and primarily affects lower motor neurons, leading to progressive muscle weakness and slow progression over time. SBMA was described in 1968 by William R. Kennedy in 11 patients from two different families, observing its X-linked recessive inheritance pattern [1]. SBMA is a trinucleotide repeat disorder caused by a CAG repeat expansion in the first exon of the androgen receptor (AR) gene, located in the Xq11-q12 region; 38 repeats are considered a full penetrance allele, while 35-37 are considered reduced penetrance alleles [2,3]. This leads to the formation of a polyglutamine tract that causes the AR to adopt abnormal conformations, resulting in protein aggregates that disrupt normal cellular function. This phenomenon is associated with neuronal toxicity and muscle degeneration [4,5].

A wide range of manifestations characterizes KD; for example, during adolescence, androgen insensitivity has been observed in the form of gynecomastia and infertility [6]. Between the ages of 20 and 30, symptoms of lower motor neuron involvement, such as weakness, cramps, and action tremors, occur [7]. Bulbar involvement follows, with fasciculations of the tongue, lips, or perioral region, dysarthria, and swallowing changes. After 10 to 20 years, individuals struggle with activities such as climbing stairs and experience muscle wasting, primarily in proximal regions and occasionally in distal regions [8]. The current prevalence of SBMA is unknown. However, it is estimated to affect approximately 1 in 40,000 men worldwide [5], with similar statistics observed in Europe, specifically in Italy, where prevalence data are available [9]. There is a higher incidence in some areas of Japan and Finland, with an approximate rate of 13 cases per 85,000 population in Finland [10].

The AR is a ligand-activated transcription factor that triggers the androgen response in target cells [5]. In the absence of ligands such as testosterone, AR remains in the cytoplasm bound to heat shock proteins (HSPs). When activated by ligands, AR translocates to the nucleus and regulates gene expression [11]. In cases of expanded polyglutamine in the AR, such as in certain neurodegenerative diseases, the receptor becomes cytotoxic, evades degradation, and forms misfolded protein aggregates. These aggregates disrupt cellular functions, including mitochondrial activity and axonal transport, leading to neuronal dysfunction and death [12], which is the basis of the progressive neuromuscular deterioration characteristic of KD [13]. The expanded CAG tract in the AR gene results in a longer polyglutamine tail, which hinders its interaction with HSPs and leads to the formation of insoluble aggregates in motor neurons [5]. The polyglutamine expansion disrupts the normal function of AR, including its role in regulating gene transcription. This leads to toxicity in the motor neuron and muscle, and ultimately to neurodegeneration [12,13]. Although CAG repeat length does not affect non-motor symptoms, it negatively correlates with the age of onset of motor symptoms [14].

This report aimed to describe two new Mexican cases of KD and to describe the clinical and molecular characteristics of all published cases in Latin America through a systematic review (SR).

## Case presentation

Case 1

A 50-year-old man with a history of type 2 diabetes mellitus (T2DM) presented with progressive weakness of the lower limbs since the age of 32 years, initially affecting the distal and later the proximal regions, with asymmetry and predominance on the left side. Since the age of 23, he has had a fine tremor in his upper limbs. This tremor increased in situations of stress. The symptoms progressed until he came to our center for increased weakness. After a few months, he reported that he was getting worse and felt more weakness when walking. His neurological examination revealed flaccid dysarthria, dysphagia, tongue atrophy, proximal weakness (4/5) in the upper limbs, decreased upper and lower limb reflexes, and facial and trunk fasciculations. These data supported the suspicion of amyotrophic lateral sclerosis (ALS), and an electromyography (EMG) was performed. The EMG revealed reduced compound muscle action potentials (CMAPs) in the upper and lower extremities. The motor nerve conduction study (NCS) showed a generalized reduction in CMAPs and normal velocities in both upper and lower extremities, with mean values of 7.1 mV (SD: 3.1) in the upper extremities and 4.3 mV (SD: 1.6) in the lower extremities, consistent with chronic axonal involvement reflecting lower motor neuron degeneration.

**Figure 1:**
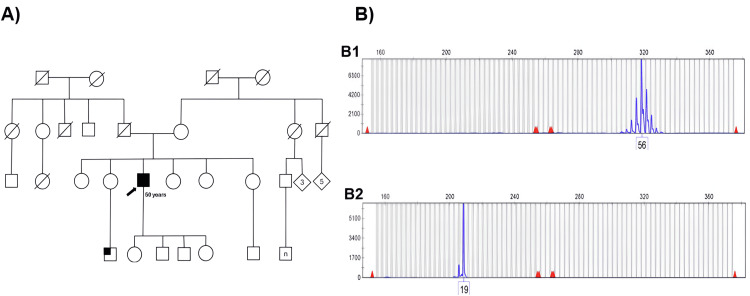
Case 1 pedigree and triplet repeat primed PCR (TP-PCR) analysis for the Kennedy disease. Image A shows the pedigree of Case 1 (arrow), and the patient's nephew with similar manifestations is marked with a small square. Image B shows the stripe pattern and a peak corresponding to 56 CAG expanded repeats in the AR gene (B1), while image B2 shows a normal allele with 19 CAG repeats. The vertical axis represents relative fluorescence units (RFU), and the horizontal axis represents allele length in base pairs (bp).

However, after several visits, gynecomastia was noted, and erectile dysfunction was confirmed to have been present for years. He also mentioned that a nephew had presented with similar symptoms but had never been followed up. Therefore, genetic analysis for CAG repeats by triplet-primed polymerase chain reaction (TP-PCR) in the AR gene was performed, and 56 CAG repeats were identified, confirming the diagnosis of KD (Figure 1). Two years after diagnosis, the exacerbated weakness in the lower extremities worsened. Physiotherapy was started, and an increase in strength in his extremities was observed; however, he reported needing manual assistance to climb stairs. He currently receives regular endocrinology consultations to manage his hyperglycemia and dyslipidemia.

Case 2

A 52-year-old male had a family history of muscular disease on his mother's side. He began experiencing lower limb weakness and facial twitching at the age of 48. Over time, he developed gait disturbance and decreased range of motion in his hips, which reduced his stride. He noticed difficulty and a sensation that his feet were "stuck," as well as increased muscle weakness, especially during physical activity, and difficulty maintaining posture, particularly at the trunk-pelvic junction. In recent years, with no improvement in his symptoms and reporting the same limitations with slow progression, the patient decided not to follow up on his symptoms. In recent months, however, he noticed worsening weakness, intermittent paresthesias, mild gynecomastia, and sexual dysfunction. Therefore, he decided to come to our center. His neurological examination revealed distal hand tremor, proximal weakness (4/5), hyporeflexia, and generalized fasciculations.

**Figure 2:**
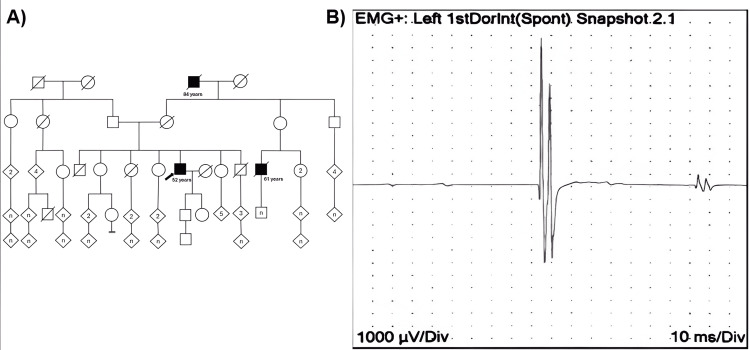
Case 2 pedigree and electromyography (EMG) showing resting fasciculation in Kennedy disease. Image A shows the pedigree of Case 2 (arrow) and other male members with suggestive Kennedy's disease. Image B shows the recording of the resting fasciculation of the first dorsal interosseous muscle in a patient with Kennedy disease. Calibration is 1000 μV per division on the vertical axis and 10 ms per division on the horizontal axis.

EMG and NCS showed moderately prolonged latencies (4.3 m/s) in both median nerves and reduced sensory conduction amplitudes. In addition, a bilateral absence of response was noted in the sural nerve. These findings were consistent with chronic axonal involvement, reflecting progressive degeneration of lower motor neurons. Furthermore, fasciculation potentials at rest indicated a loss of muscle innervation (Figure 2), whereas incomplete recruitment patterns during maximal contraction suggested progressive motor unit degeneration. The absence of bilateral sural nerve responses also indicated severe sensory neuropathy, suggesting significant sensory involvement.

Given the uncertainty of the diagnosis, genetic testing by TP-PCR identified 52 CAG repeats in the AR gene, confirming KD. Sometime after the diagnosis, the patient presented with dysarthria. The patient's current treatment includes physical, communication, and swallowing therapy; pregabalin; and electrophysiologic therapy for erectile dysfunction. After the diagnosis, the patient gathered more information from the deceased family members who had the muscle disease and confirmed that they had experienced the same symptomatology.

Systematic review search

**Figure 3:**
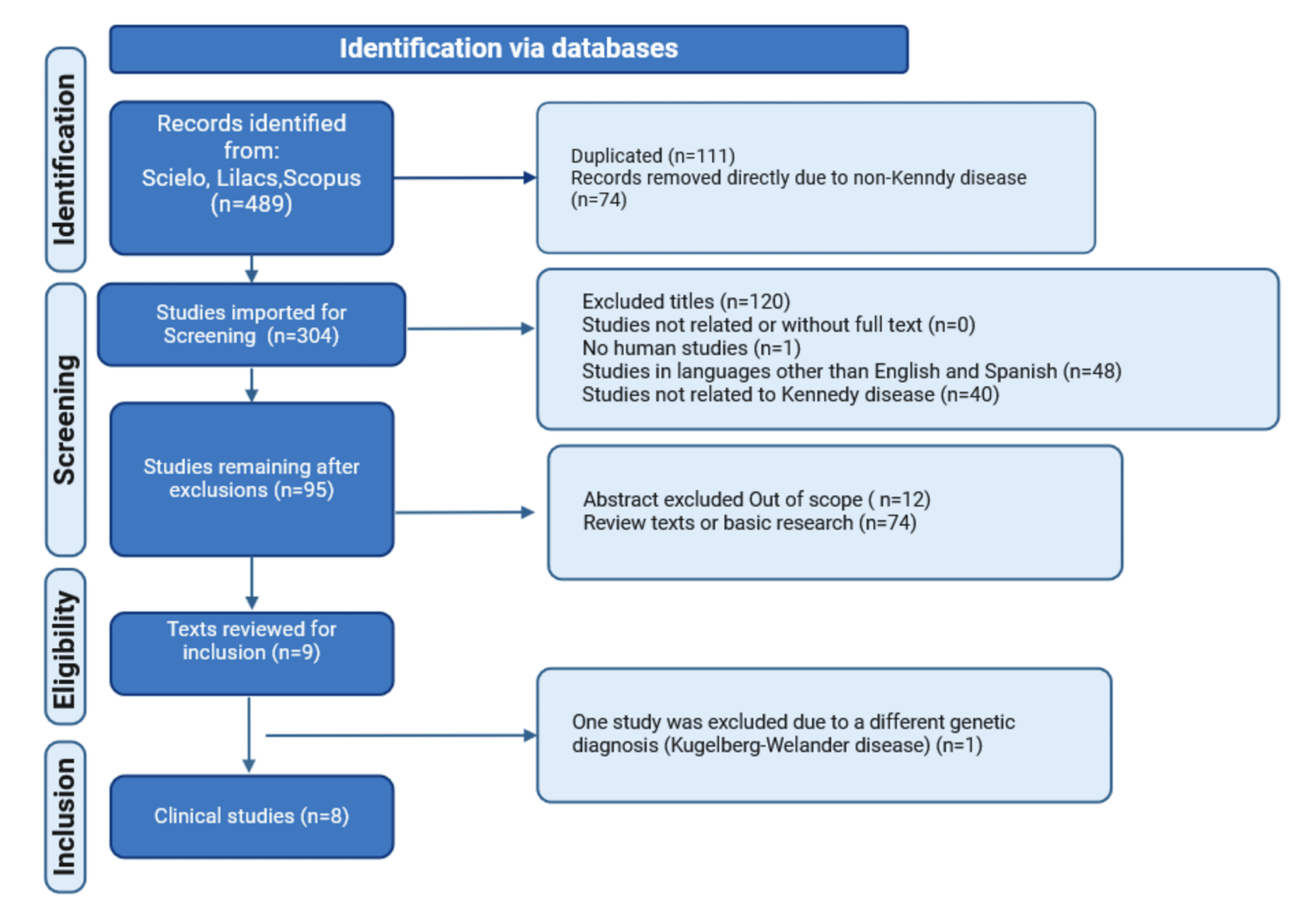
Flowchart of the systematic review search strategy.

A literature search was performed using the Preferred Reporting Items for Systematic Reviews and Meta-Analyses (PRISMA) method. Although the boundaries of Latin America are often disputed, it is generally accepted that it consists of Mexico, Central America, all of South America, and the islands of the Caribbean whose inhabitants speak a Romance language. The Latin American databases Scielo and Lilacs were searched from 1991 to May 2024. The combination of the terms "Kennedy disease", "spinal and bulbar muscular atrophy", and "AR gene" was used for English and for each language (Spanish: "Enfermedad de Kennedy", "atrofia muscular espinal y bulbar", and "gen AR"; and Portuguese: "Doença de Kennedy", "Atrofia muscular spinal e bulbar", and "gene AR"). We also performed a search in the Scopus database using the keywords "Kennedy disease", "spinal and bulbar muscular atrophy", "AR gene", and "Latin America", limiting the search to the available Latin American countries (Argentina, Brazil, Colombia, Honduras, Mexico, Panama, Peru, and Puerto Rico). The strategy of the SR search is shown in Figure 3.

The search was performed by two authors (C.A.M.-Z. and S.U.V.-M.) who independently reviewed the articles. In case of disagreement, a third author (M.A.R.-G.) was consulted to resolve the conflict and to ensure that all included studies met the criteria. We included all publications (cohorts, case series, case reports, or editorials) describing the phenotype of interest with molecular confirmation (selection criteria). We excluded: (1) articles that did not focus on the phenotype of interest, (2) animal or in vitro studies, or (3) texts that were not available. Information extracted included author, year of publication, age of onset, neuromuscular symptoms, bulbar symptoms, androgen deficiency symptoms, ancillary studies, molecular results, and other findings.

Systematic review of published cases in Latin America

Through eight reports and the two cases described above, the review identified 23 cases from Latin America by May 2024. The median age of onset was 43 years (range 21-66). Weakness was the most common symptom, affecting 95.7% (n = 22) of cases, 65.2% (n = 15) with tremor, and 69.5% (n = 16) with fasciculations. Bulbar involvement, such as dysarthria and dysphagia, was found in 95.6% (n = 22) and 91.3% (n = 21), respectively. Meanwhile, androgen deficiency, such as gynecomastia, was seen in 73.9% (n = 17), and sexual dysfunction in 56.5% (n = 13). Electrophysiological changes compatible with KD were observed in all subjects. Molecular analysis revealed a median number of CAG repeats of 46.5 (range 35-56). A correlation between age at onset and CAG repeat length showed a correlation coefficient of r = -0.042. Additional clinical findings are described in the Appendices.

## Discussion

KD is a rare genetic neuromuscular disorder with an estimated prevalence of 1 in 40,000 worldwide. However, prevalence estimates vary, from about 1 in 6,500 in Finland to 1 in 30,000 in regions of Italy [10,22]. Even in the United Kingdom, despite the establishment of a national registry for the disease, only 61 patients were identified between 2005 and 2013 [14]. Our SR, which focused on Latin America, identified only 23 cases of KD (including the two cases of this report).

KD has a highly variable phenotype, but neuromuscular, androgen deficiency, and bulbar involvement are the most representative manifestations. In most cases, weakness and neuromuscular symptoms are the main reasons for seeking medical attention, as was the case in our patients. It can present between the ages of 21 and 66 years, and in Latin American cases, the median age of onset was similar to that in another region [23]; 95.7% (n = 22) presented with weakness, 65.2% (n = 15) with tremor, and 69.5% (n = 16) with fasciculations. According to the largest cohort of KD, the expected course of the disease shows that muscle weakness begins in middle age (45 years), with the need for a wheelchair occurring between 15 and 20 years later (around 61 years) [24]. The involvement of motor neurons in the medulla oblongata is associated with symptoms such as dysphagia, dysarthria, facial weakness, and voice changes, typically beginning in the fifth decade of life and affecting about 90% of cases [25]. In Latin American cases, the proportion of bulbar involvement was similar, with dysarthria being the most common, followed by dysphagia and tongue fasciculations. The manifestations of androgen deficiency are diverse, and the frequencies of gynecomastia (73.9%, n = 17), sexual dysfunction (56.5%, n = 13), and infertility (78.2%, n = 18) found in Latin American cases are similar to those in another report [26].

On the other hand, there is a high prevalence of metabolic and liver disorders in KD patients. In these patients, abnormal plasma lipid levels, insulin resistance or diabetes, and non-alcoholic fatty liver disease have been reported [13], which have been attributed to mitochondrial dysfunction and altered metabolic pathways in the affected tissues. In Latin American cases, there was a lack of information in this regard, and only 13.0% were described as having T2DM. However, the prevalence of comorbidity with T2DM in KD is unknown [27].

In terms of differential diagnosis, neuromuscular and bulbar involvement may lead to confusion with other entities. Approximately 10% of patients are initially misdiagnosed as having ALS, as was the case in our patients. ALS is a neurodegenerative disease that also affects motor neurons in the brain, brainstem, and spinal cord, resulting in progressive atrophy and paralysis of voluntary muscles [28]. However, the progression is faster and more aggressive, is not associated with androgenic dysfunction, and does not involve lower motor neurons. On the other hand, when evaluating a patient with KD (weakness, fatigue, especially when accompanied by fasciculations, muscle atrophy, or areflexia), another common misdiagnosis is myasthenia gravis (MG). MG is an autoimmune disease characterized by variable muscle weakness and fatigue associated with impaired neuromuscular transmission. MG can present with facial, eye, and limb weakness, as well as with dysarthria and dysphagia, and may respond well to pharmacologic treatment [29].

Because of this overlap in the diagnostic approach, molecular evaluation of SBMA is essential to establish the diagnosis. However, underdiagnosis may also be associated with reduced penetrance in carriers of shorter CAG repeats (35-37) [30]. In our series, patients had CAG repeat lengths ranging from 35 to 56 and showed a low correlation with age of onset (r = -0.042), in contrast to what has been described in other series [31], which could be explained by the small sample size. A recent study showed that CAG repeat expansions in the AR gene are common in the general population, suggesting potential underdiagnosis or reduced penetrance [32]. In addition, due to its X-linked inheritance, it is essential to provide appropriate genetic counseling and to explain the role of female carriers, who are often asymptomatic but may transmit the disease [33]. However, no female patients have been reported in Latin America.

Management of patients with KD is primarily symptomatic and supportive. While life expectancy is generally within the normal range, certain cases may be severe and potentially fatal due to complications such as aspiration pneumonia or respiratory problems associated with bulbar weakness. In addition to neuromuscular evaluation, cardiac and hepatic assessment is necessary [13], as well as annual testing of lipid levels, cholesterol, and blood glucose [27]. Hormone replacement therapy with testosterone has historically been a treatment option for KD, as it is postulated that increased testosterone levels may counteract this deficiency and avert muscular atrophy [34]. Therefore, new drugs such as AR modulators are being investigated to restore normal androgen signaling [35]. Dimethylcurcumin, or ASC-J9, has been investigated for its ability to interfere with the aberrant AR and its co-regulators, apparently promoting its degradation and mitigating the toxicity associated with the accumulation of mutant AR in cells. Even an Hsp90 inhibitor has been evaluated [36,37]. Physiotherapy is needed to maintain mobility and prevent contractures, while nutritional support is crucial in the context of dysphagia [38]. In addition, psychological and genetic counseling are needed to help patients and their families cope with the emotional burden of the disease [39].

Despite its low prevalence, there is little published information on KD in Latin America. This scarcity may be due in part to a lack of recognition of the pathology and the fact that epidemiology is still at an early stage in the region. It also suggests limited interest in establishing a casuistry of the disease. However, this report represents the first situational approach to KD in Latin America through an SR using the Scopus, Scielo, and Lilacs databases. Therefore, further research on this disease is essential to develop protocols and management guidelines in the region.

## Conclusions

The study and increased clinical awareness of KD in Latin America are essential for advancing understanding of its regional epidemiology and improving recognition of its classical manifestations, namely, neuromuscular, bulbar, and endocrine involvement. Moreover, as KD is increasingly recognized as a multisystem disorder, attention should also be directed toward less commonly evaluated domains, including the cardiac, metabolic, skeletal, and genitourinary systems. Strengthening diagnostic protocols, promoting early genetic testing, and integrating multidisciplinary management strategies are critical steps toward comprehensive care. Additionally, the development of region-specific clinical guidelines and the implementation of accessible genetic counseling services are vital to address underdiagnosis and improve outcomes for affected individuals across Latin America.

## Appendices

Reported cases of Kennedy disease in Latin America are provided in the following link: https://docs.google.com/spreadsheets/d/1qiIhwI7Yc5P5hukCpxOzpxu5AH7zL4I6/edit?gid=1170435322#gid=1170435322. In the dataset, X indicates the presence of a manifested symptom, N indicates that the symptom was not manifested, and (-) indicates that data are not available.
